# Matching-Adjusted Indirect Comparison of the Efficacy and Safety of Erdafitinib vs Enfortumab Vedotin in Patients with Locally Advanced Metastatic Urothelial Carcinoma

**DOI:** 10.36469/001c.120954

**Published:** 2024-08-20

**Authors:** Suzy Van Sanden, Ayman Youssef, Simona Baculea, Keith Stubbs, Spyros Triantos, Zijiao Yuan, Caitlin Daly

**Affiliations:** 1 Janssen Pharmaceutica NV, Beerse, Belgium; 2 Janssen Cilag, Issy-les-Moulineaux, France; 3 Janssen Cilag Ltd, High Wycombe, United Kingdom; 4 Johnson & Johnson, Spring House, Pennsylvania, United States; 5 Cytel Inc., Rotterdam, Netherlands; 6 Cytel Inc., Toronto, Ontario, Canada

**Keywords:** locally advanced unresectable metastatic urothelial carcinoma, erdafitinib, enfortumab vedotin, indirect treatment comparison, matching-adjusted indirect comparison

## Abstract

**Background:** For patients with locally advanced or metastatic urothelial carcinoma (la/mUC), prognosis is poor and effective treatment options are limited. Erdafitinib is an oral fibroblast growth factor receptor (FGFR) kinase inhibitor approved by the FDA for the treatment of adults with la/mUC harboring FGFR alterations whose disease progressed following at least 1 prior line of therapy, including a PD-1 or PD-L(1) inhibitor, based on the phase 3, randomized THOR trial (NCT03390504, Cohort 1).

**Objective:** To compare the efficacy and safety of erdafitinib vs enfortumab vedotin-ejfv (EV) in the absence of head-to-head comparison via an anchored matching-adjusted indirect comparison (MAIC).

**Methods:** An anchored MAIC was conducted according to the National Institute for Health and Care Excellence Decision Support Unit guidance, with physician’s choice of chemotherapy (docetaxel/paclitaxel and vinflunine) as the common comparator. Individual patient data from THOR were adjusted to match published key eligibility criteria and average baseline characteristics of EV-301, such as Bellmunt risk score, liver or visceral metastases, primary site, among others. Erdafitinib was then indirectly compared with EV using the relative treatment effects for the reweighted THOR population and those published for EV-301.

**Results:** After matching, the effective sample size for THOR was 126 patients. The MAIC-recalculated hazard ratio (95% credible interval) for erdafitinib vs EV was 0.92 (0.54, 1.57) for overall survival and 0.93 (0.55, 1.56) for progression-free survival, yielding Bayesian probabilities of erdafitinib being better than EV of 62.1% and 60.5%, respectively. For response outcomes, the MAIC-recalculated risk ratio was 1.49 (0.56, 3.90) for confirmed objective response rate and 2.89 (0.27, 30.33) for confirmed complete response with probabilities of 72.6% and 81.3% for erdafitinib being better than EV, respectively. For safety, MAIC-yielded risk ratios of 1.09 (0.99, 1.21) for any treatment-related adverse events, 0.86 (0.57, 1.28) for grade 3+ TRAEs, and 1.02 (0.98, 1.06) for any treatment-emergent adverse events.

**Conclusion:** The MAIC indicates comparable efficacy of erdafitinib vs EV for overall survival and progression-free survival, with erdafitinib showing a higher probability of achieving deep responses. While erdafitinib is associated with slightly more adverse events compared with EV, these events seem to be less severe.

## INTRODUCTION

Bladder cancer is the ninth most common malignancy globally, with 614 298 cases diagnosed and 220 596 deaths reported in 2022.^1^ Approximately 90% of bladder cancer cases are urothelial carcinoma, which arises from the transitional cells in the lining of the bladder, ureter, or kidney.^2^

Urothelial carcinoma poses a significant clinical challenge due to its aggressive nature and propensity for local invasion and metastasis.^2,3^ Locally advanced or metastatic urothelial carcinoma (la/mUC) is considered incurable with a dismal prognosis.^4^ Historically, standard first-line treatment for patients with la/mUC has generally been cisplatin-based combination chemotherapy for those who are eligible or carboplatin-based combinations for those ineligible.^5–7^ More recently, data supports the use of combination regimens including immune checkpoint inhibitors (ICIs) if PD(L)-1 expression is established, with enfortumab vedotin-ejfv (EV) in combination with pembrolizumab being the preferred treatment in the first-line setting.^5–7^ With standard chemotherapy options, reported overall survival (OS) for later-line patients is less than 9 months, while the majority of patients receiving ICIs in this setting fail to achieve a complete or durable response to treatment.^8^ Erdafitinib and EV are among the novel treatment options for patients with la/mUC who progress following chemotherapy and/or anti-PD(L)-1 treatment.^5–7^

Erdafitinib is an oral fibroblast growth factor receptor (FGFR) kinase inhibitor approved by the Food and Drug Administration (FDA) for the treatment of adults with la/mUC containing susceptible FGFR3 genetic alterations that have progressed during or following at least 1 prior line of systemic therapy, that included a PD-1 or PD-L(1) inhibitor if eligible.^9,10^ The safety and efficacy of erdafitinib in patients with la/mUC containing FGFR aberrations was investigated in the phase 3, randomized THOR trial (NCT03390504, Cohort 1). At a median follow-up time of 15.9 months, treatment with erdafitinib demonstrated statistically significant improvement vs chemotherapy (docetaxel or vinflunine) in terms of OS (hazard ratio [HR], 0.64; 95% confidence interval [CI] 0.47, 0.88), and progression-free survival (PFS) (HR 0.58; 95% CI, 0.44, 0.78).^11^

EV is an antibody drug conjugate directed against the adhesion protein nectin-4, which is expressed on the surface of urothelial carcinoma cells.^12^ It is approved by both the FDA and the European Medicines Agency to treat adults with la/mUC who have previously undergone platinum-based therapy and have been treated with a PD(L)-1 inhibitor.^13,14^ Additionally, in the United States, EV is approved for the treatment of cisplatin-ineligible patients with la/mUC who have received prior therapy or in combination with pembrolizumab regardless of prior treatment.^15^ The safety and efficacy of EV was evaluated in an open-label, randomized, phase 3, multicenter study (EV-301, NCT03474107) in patients with la/mUC who had previously received platinum-containing chemotherapy and a PD(L)-1 inhibitor.^12,16^ Compared with chemotherapy (docetaxel, paclitaxel, or vinflunine), EV demonstrated a significant extension in OS (HR, 0.70; 95% CI, 0.56, 0.89) and PFS (HR, 0.62; 95% CI, 0.51, 0.75) following 11.1 months of median follow-up.^12^

To date, no trials have directly compared erdafitinib with EV. In the present analysis, we used a matching-adjusted indirect treatment comparison (MAIC) to explore the relative efficacy and safety of erdafitinib for the treatment of patients with la/mUC whose disease has progressed after 1 or 2 prior treatments, including an anti-PD(L)-1 in the THOR trial vs EV in EV-301.

## METHODS

### Data Sources and Study Characteristics

This analysis used individual patient data from THOR (Cohort 1) and published aggregate data from EV-301.^12^ Both trials were broadly comparable regarding comparator, trial design, and outcomes (**Tables S1 and S2**). There were, however, some differences. THOR enrolled adults with metastatic or surgically unresectable UC who had at least 1 FGFR3 alteration, whereas EV-301 enrolled adults with metastatic or unresectable locally advanced UC regardless of genetic alteration status.^11^ Patients enrolled in THOR previously received up to 2 prior systemic therapies including an anti-PD(L)-1 therapy in any setting, without a specific requirement for platinum-based therapy. For EV-301, patients were required to have received up to 3 prior lines of systemic therapy, including an anti-PD(L)-1 agent, and a platinum-based therapy in any setting with the condition of no more than 1 prior chemotherapy regimen in the locally advanced or metastatic disease setting.^12^ Patients in THOR had an Eastern Cooperative Oncology Group performance status (ECOG PS) score of 0 to 2, whereas patients in EV-301 had ECOG PS of 0 to 1. No other differences were observed in the key eligibility criteria between the two trials.

The intention-to-treat (ITT) population in THOR (Cohort 1) consisted of 266 patients, of whom 136 were randomized to the erdafitinib arm, and 130 were randomized to physician’s choice of chemotherapy; the safety population included 135 patients in erdafitinib and 112 patients in chemotherapy group, accounting for all randomized subjects who received at least 1 dose of trial drug.^11^ The ITT population in EV-301 was 608 patients, with 301 in the EV arm and 307 in the physician’s choice of chemotherapy arm; the safety population in EV-301 included patients who received any amount of drug, with 296 in the EV arm and 291 in the chemotherapy arm.^12^

The median follow-up for THOR was 15.9 months,^11^ whereas EV-301 had a median follow-up of 11.1 months at the interim analysis^12^ and 23.75 months at the longer-term analysis.^16^

### Common Comparator

In THOR and EV-301, the common comparator arm was physician’s choice of chemotherapy with vinflunine or docetaxel. EV-301 also allowed the additional choice of paclitaxel in the chemotherapy arm (**Table S3**).^11,12^ Docetaxel and paclitaxel, which have reported median OS of 9.0^17^ and 7.2^18^ months, respectively, were assumed to be equivalent in terms of efficacy as per individual clinician consultation, therefore, “physician’s choice of chemotherapy” in both trials was considered sufficiently comparable to be considered a common comparator.

### Outcomes

The comparative efficacy of erdafitinib vs EV was determined for OS, PFS, confirmed overall response rate (cORR), and confirmed complete response (cCR) rate. For comparative safety, treatment-related adverse events (TRAE) and treatment-emergent adverse events (TEAE) were considered. Endpoint definitions were generally similar between THOR and EV-301 (**Table S4**). Both trials used RECIST 1.1 criteria to determine disease progression and responses. However, the timing of the assessments was every 6 weeks ± 7 days for THOR for the first year, and as clinically indicated afterwards, while the timing of the assessments was every 8 weeks ± 7 days for EV-301. With respect to safety, both trials graded adverse events (AEs) according to CTCAE v4.03.

### Statistical Analysis

Anchored MAICs between erdafitinib and EV using individual patient data from THOR and published aggregate data from EV-301 were conducted according to guidance from the National Institute for Health Care and Excellence (NICE) Decision Support Unit (DSU) *Technical Support Document (TSD) 18*.^19^ The adjusted relative effects in THOR were computed in SAS 9.4. (SAS Institute Inc.), while the indirect treatment comparisons were conducted in WinBUGS.

As a first step, the eligibility criteria of THOR were aligned to those of EV-301 by excluding patients from THOR who had an ECOG PS of 2, no prior platinum-based chemotherapy, and/or receipt of more than 1 prior line of chemotherapy and therefore did not meet the inclusion criteria of EV-301. In addition, for the analyses of response endpoints, patients enrolled in THOR who were not considered response-evaluable according to clinical expert-validated criteria at baseline (defined as patients without target lesions at baseline) were excluded to align with the approach used in EV-301. Patients who were not considered as evaluable at follow-up were excluded from both trials. Baseline characteristics were not available for the response-evaluable population specifically; therefore, matching was only possible for the overall population and the assumption was made that the distribution of baseline characteristics in the EV-301 ITT population was similar to that of the response-evaluable population.

Next, potential differences in effect modifiers were addressed by matching the patient characteristics from the remaining THOR patient population to the reported aggregate baseline EV-301 data. The weights are propensity scores, which predicted whether a given type of patient originates from THOR or EV-301 as a function of baseline characteristics, estimated using the method of moments as described by Signorovitch et al.^20^ Clinically relevant characteristics that were adjusted for were selected via literature review and consultation with clinical experts as well as whether the characteristics were reported in both trials. The characteristics included in the matching process were Bellmunt risk score; ECOG PS 0 or 1; presence of liver metastases; presence of visceral metastases; origin of primary disease (upper urinary tract, bladder, or other site); smoking status; history of diabetes or hyperglycemia; geographic region (Western Europe, United States, rest of the world); median age (≥75 years, <75 years); and male sex.

### Adjusted Relative Effects Calculations

The individual patient weights were used to calculate adjusted relative effects for patients in THOR, reflecting the estimated impact of erdafitinib vs chemotherapy within the patient population of EV-301 trial. Binary outcomes in THOR were analyzed using a weighted logistic regression model, from which both the odds ratios (OR) and response/risk ratios (RR) were derived. Time-to-event outcomes were analyzed using a weighted Cox proportional hazards model, from which the HRs were derived. The standard errors of the relative effects were computed using a robust sandwich estimator.^21^

### Indirect Comparison via Bayesian Network Meta-analysis Methodology

The adjusted relative effects in THOR were compared with the observed relative effects of EV vs chemotherapy in EV-301 (ie, log-HRs for PFS and OS, log-ORs for cORR, cCR, and safety outcomes) to estimate the comparative efficacy of erdafitinib vs EV using the methodology of the Bayesian network meta-analysis (NMA) with the special case of an NMA with only 2 studies. Fixed-effects NMA models were fitted using Monte Carlo Markov Chain simulation methods, following the methods described in NICE DSU TSD 2.^22^ Noninformative normal (0, 1002) priors were assigned to the basic relative effect parameters. All models were run using three chains with a burn-in period of 50 000 iterations.

Convergence was assessed using the Brooks-Gelman-Rubin diagnostic and history plots.^23,24^ A further simulation sample of 50,000 iterations for each chain was used to inform the results. For all outcomes, relative effects and their 95% credible intervals (CrI) of erdafitinib vs EV were estimated (for binary outcomes, ORs and RRs; for survival outcomes, HRs). To visualize time-to-event outcomes, Kaplan-Meier curves for the adjusted THOR population were superimposed with the observed curves for the EV-301 population.

### Assessment of Proportional Hazards

The MAIC of erdafitinib vs EV for time-to-event outcomes assumed proportional hazards held for the comparisons of erdafitinib vs chemotherapy within the matched THOR data and EV vs chemotherapy within the EV-301 data. To assess this assumption, we visually inspected the log-cumulative hazards for each treatment group and Schoenfeld residuals plots. The Grambsch and Therneau test was also conducted to quantitatively assess whether evidence suggesting violation of the proportional hazards assumption was present.^25^

### Sensitivity Analyses

Several sensitivity analyses were conducted for the MAIC in which (1) each baseline characteristic was cumulatively adjusted for one-by-one in order of their clinical relevance, (2) PFS and OS data from the subgroup in EV-301 who had received only 1 or 2 prior lines of treatment were used, and (3) data from the longer-term follow-up in EV-301 (median follow-up of 23.75 months^16^ were used.

The sensitivity analysis that used the subgroup receiving 1 or 2 prior treatments in EV-301 was conducted because it was not feasible to match patients with 3 or more lines of treatment in EV-301’s ITT population, since nearly all patients in THOR received up to 2 prior treatments. However, this analysis was limited due to unavailable baseline characteristics in this subgroup in EV-301; patients in THOR could only be matched to EV-301’s ITT population. The underlying assumption implicitly made for this analysis is that the distribution of baseline characteristics in the 1-2 prior treatment subgroup was the same as those in the ITT population in the EV-301 trial.

Additionally, EV-301 reported outcomes at an interim analysis with a median follow-up of 11.1 months, and in the longer-term with a median follow-up of 23.75 months. Thus, the base-case analysis was informed by EV-301’s interim data, as the corresponding follow-up was more similar to THOR’s median follow-up (15.9 months). The impact of differences in follow-up time were assessed in a sensitivity analysis using the longer-term data in EV-301.

## RESULTS

Prior to matching, the patient populations in THOR and EV-301 at baseline differed with respect to Bellmunt risk scores, liver metastases, history of diabetes or hyperglycemia, geographic region, and sex, whereas the proportions of patients who were at least 75 years old, were nonsmokers, had primary disease originating in the urinary tract, and had visceral metastasis were generally comparable (**Table 1**). Compared with EV-301, THOR had a slightly larger proportion of patients with Bellmunt risk scores of 0-1 (74% vs 68%; standardized mean difference [SMD]: 0.13), lower proportion of males (71% vs 77%; SMD, −0.14) and history of diabetes or hyperglycemia (12% vs 19%; SMD, −0.19). THOR also had a significantly higher proportion of patients from the Western Europe (61% vs 42%; SMD: 0.39).^12^ Full patient demographics and baseline characteristics for THOR and EV-301 are presented in **Table S5**.

Baseline characteristics ranked in order of clinical significance as determined by physician expert consultation and characteristics which were not able to be fully adjusted are provided in **Table S6** and **Table S7**, respectively. For the analysis, a total of 69 of the 266 patients were excluded from THOR because they had an ECOG PS of 2 (n = 25), no prior platinum-based chemotherapy (n = 33), or more than one prior chemotherapy (n = 11). Following matching, the baseline characteristics of patients in the two studies were well balanced (**Table 1**). The effective sample size (ESS) for THOR decreased from 197 to 126 patients, a reduction of 36%.

**Table 1.** Demographic and Baseline Characteristics of EV-301 and THOR Before and After Matching

	**EV-301 (N=608)**	**THOR (N=266)**	**THOR** **Exclusion Criteria Applied (n = 197)**	**THOR** **Matched (n = 197, ESS = 126)**
Bellmunt risk score (%)				
0-1	68	74	78	68
2	32	26	22	32
ECOG PS (%)				
0	40	43	48	40
1	60	48	52	60
Presence of liver metastasis (%)	25	26	26	25
Presence of visceral metastasis (%)	66	74	76	66
Origin of primary disease (%)				
Upper urinary tract	34	33	35	34
Bladder or other site	66	67	65	66
Smoking status (%)				
Never smoked	33	34	31	33
History of diabetes or hyperglycemia (%)	19	12	12	19
Region (%)				
Western Europe	42	61	60	42
United States	14	5	5	14
Other	44	34	36	44
Age, y (%)				
<75	68	67	67	68
≥75	20	21	20	20
Sex, male (%)	77	71	71	77

Abbreviations: ECOG PS, Eastern Cooperative Oncology Group performance score; ESS, effective sample size, SMD, standardized mean difference.

### Overall Survival and Progression-Free Survival

**Figure 1.**
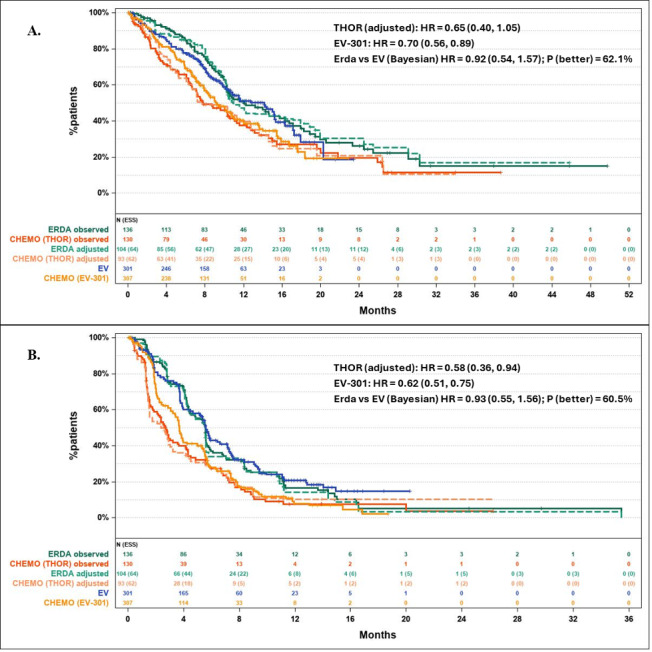
Survival Results for Erdafitinib vs EV **(A)** Kaplan-Meier curves for overall survival for ERDA and CHEMO arms (observed and adjusted) from THOR and EV and CHEMO arms from EV-301. **(B)** Kaplan-Meier curves for progression-free survival for ERDA and CHEMO arms (observed and adjusted) from THOR and EV and CHEMO arms from EV-301. Adjustment had limited impact on the relative effectiveness and OS and PFS of ERDA and EV were comparable. Abbreviations: CHEMO, chemotherapy; ERDA, erdafitinib; EV, enfortumab vedotin; HR, hazard ratio.

In terms of OS and PFS, the impact of matching within the THOR study was minimal (**Figure 1, Table 2**). Cumulative analyses, starting with matching only one baseline characteristic and then cumulatively adding additional baseline characteristics showed that some of them had impact (eg, ECOG, liver and visceral metastasis), yet this was counterbalanced by opposite effects from others (eg, sex and history of diabetes or hyperglycemia), leaving the HR unchanged (**Figures S1 and S2**). Consequently, the HRs of erdafitinib vs EV were similar before and after matching.

**Table 2.** Survival Outcomes for Erdafitinib vs EV

	**HR (95% CI)**		**HR (95% CrI)**	**Probability Erdafitinib Is Better than EV (%)**
	**EV-301: EV vs** **Chemotherapy**	**THOR: Erdafitinib vs Chemotherapy**	**MAIC: Erdafitinib vs EV**	
OS				
Observed	0.70 (0.56, 0.89)	0.64 (0.47, 0.88)	0.91 (0.62, 1.35)	68.0
Exclusion criteria applied	–	0.65 (0.44, 0.94)	0.92 (0.59, 1.43)	64.1
Matched	–	0.65 (0.40, 1.05)	0.92 (0.54, 1.57)	62.1
PFS				
Observed	0.62 (0.51, 0.75)	0.58 (0.44, 0.78)	0.94 (0.67, 1.33)	63.9
Exclusion criteria applied	–	0.54 (0.38, 0.75)	0.87 (0.59, 1.28)	76.4
Matched	–	0.58 (0.36, 0.94)	0.93 (0.55, 1.56)	60.5

Abbreviations: CI, confidence interval; CrI, credible interval; EV, enfortumab vedotin; HR, hazard ratio; MAIC, matching adjusted indirect comparison; OS, overall survival; PFS, progression-free survival.

Erdafitinib had a postadjustment OS HR of 0.92 (95% CrI: 0.54, 1.57) compared with EV, and a 62.1% probability of being better than EV (**Figure 1A**). With respect to PFS, the postadjustment HR of erdafitinib vs EV was 0.93, (95% CrI: 0.55, 1.56), with a 60.5% probability of erdafitinib being better than EV (**Figure 1B**). The posterior distributions of the HRs of OS and PFS between erdafitinib and EV are provided in **Figures S3A and S3B**. The Bayesian probability is visually represented as the area under the distribution curve to the left of HR = 1.

For both OS and PFS, the sensitivity analyses were generally consistent with the base case. For the analysis in which each baseline characteristic was cumulatively adjusted one-by-one, the MAIC estimates for the HR of erdafitinib vs EV were consistent across all baseline characteristics (**Figures S1 and S2**). The results of the analysis of OS and PFS for the subgroup who had received 1 or 2 prior lines of therapy were similar to the main analysis (**Table S8**). The longer-term (23.75 months) OS and PFS results in EV-301 were also consistent with the interim results (11.1 months) (**Table S9**).

### Response Rates

After matching, a small reduction in the cORR rates for erdafitinib and chemotherapy in the THOR trial was noted, decreasing from 38.5% and 13.8% to 37.0% and 11.7% (**Table 3**). The cCR rate of erdafitinib improved minimally from 3.4% to 3.6%, whereas the cCR rate for chemotherapy decreased from 1.3% to 0.7%; **Table 3**). These changes resulted in an increased RR of erdafitinib vs chemotherapy after matching.

**Table 3.** Response Outcomes for Erdafitinib vs EV

	**EV-301**			**THOR**			**MAIC**		
	**Response Rate EV/Chemo**	**OR: EV/Chemo (95% CI)**	**RR: EV/Chemo (95% CI)**	**Response Rate Erdafitinib/Chemo**	**OR: Erdafitinib/ Chemo (95% CI)**	**RR: Erdafitinib/ Chemo (95% CI)**	**OR** **(95% CrI)**	**Probability Erdafitinib Is Better Than EV (%)**	**RR (95% CrI)**
cORR									
Observed	46.6%/22.0%	3.10 (2.09, 4.59)	2.12 (1.61, 2.78)	38.5%/13.8%	3.92 (1.88, 8.19)	2.80 (1.54, 5.09)	1.27 (0.55, 2.91)	71.0	1.32 (0.68, 2.54)
Exclusion criteria applied	–	–	–	41.1%/12.5%	4.89 (2.09, 11.45)	3.29 (1.63, 6.62)	1.58 (0.61, 4.01)	82.9	1.55 (0.73, 3.26)
Matched	–	–	–	37.0%/11.7%	4.43 (1.48, 13.30)	3.16 (1.25, 7.99)	1.43 (0.44, 4.56)	72.6	1.49 (0.56, 3.90)
cCR									
Observed	5.6%/3.3%	1.72 (0.71, 4.18)	1.68 (0.72, 3.94)	3.4%/1.3%	2.80 (0.31, 25.49)	2.73 (0.31, 24.34)	1.62 (0.15, 17.24)	65.4	1.62 (0.15, 16.66)
Exclusion criteria applied	–	–	–	5.5%/1.5%	6.71 (0.76, 59.35)	6.43 (0.74, 55.97)	3.88 (0.36, 40.33)	87.1	3.80 (0.37, 38.36)
Matched	–	–	–	3.6%/0.7%	5.03 (0.56, 45.10)	4.89 (0.54, 44.36)	2.91 (0.27, 30.57)	81.3	2.89 (0.27, 30.33)

Abbreviations: CI, confidence interval; cCR, confirmed complete response; cORR, overall response rate; CrI, credible interval; EV, enfortumab vedotin; OR, odds ratio; RR, risk ratio.

The postadjustment RR of erdafitinib vs EV for cORR was 1.49 (95% CrI: 0.56, 3.90), with 72.6% probability of erdafitinib being better than EV. For cCR, the post-adjustment RR was 2.89 (95% CrI: 0.27, 30.33), with 81.3% probability of erdafitinib being better than EV. The posterior distributions of the ORs for cORR and cCR between erdafitinib and EV are provided in **Figure S3, C and D**, and the area under the distribution curve to the right of OR = 1 indicates the probability of erdafitinib being better than EV.

In the sensitivity analyses where each baseline characteristic was cumulatively adjusted one-by-one, the MAIC estimates of erdafitinib vs EV were similar across all baseline characteristics for cORR and cCR (**Figures S4 and S5**). The cORR and cCR at 23.75 months follow-up in EV-301 closely resembled the interim findings (11.1 months)(**Table S9**).

### Safety

The relative safety of erdafitinib vs EV and the specific AE rates before and after matching are presented in **Table 4**. For any TRAE, the probability of erdafitinib being better than EV was 0.8%; however, the associated RR was 1.09 (95% CrI: 0.99, 1.21). For grade 3+ TRAEs, the probability of erdafitinib being better than EV was 77.9%, with an RR of 0.86 (95% CrI: 0.57, 1.28). For any TEAE, the probability of erdafitinib being better than EV was 13.0% and the RR was 1.02 (95% Crl: 0.98, 1.06). For the remaining TEAEs, ORs and RRs were roughly 1. The results of the sensitivity analysis that used the 23.75-month follow-up data from the EV-301 trial were similar to the base-case analysis (**Table S9**). Additionally, an exploratory analysis of additional treatment-related safety outcomes that were only reported in the 23.75-month follow-up data yielded probabilities of erdafitinib being better than EV of 88.0% for any serious TRAE, 83.0% for any TRAE leading to treatment withdrawal, and 83.8% for any TRAE leading to death (**Table S10**).

**Table 4.** Safety Outcomes for Erdafitinib vs EV

	**EV-301**			**THOR**			**MAIC**		
	**Event rate: EV/Chemo**	**OR: EV/Chemo (95% CI)**	**RR: EV/Chemo (95% CI)**	**Event Rate: Erdafitinib/Chemo**	**OR: Erdafitinib/Chemo (95% CI)**	**RR: Erdafitinib/Chemo (95% CI)**	**OR (95% CrI)**	**Probability Erdafitinib Is Better Than EV**	**RR** **(95% CrI)**
Any TRAE									
Observed	93.9%/91.8%	1.39 (0.74, 2.62)	1.02 (0.98, 1.07)	97.0%/86.6%	5.06 (1.63, 15.74)	1.12 (1.04, 1.21)	3.64 (0.99, 13.33)	2.6	1.09 (1.00, 1.20)
Exclusion criteria applied	–	–	–	98.1%/88.4%	6.64 (1.41, 31.22)	1.11 (1.02, 1.20)	4.77 (0.89, 25.18)	3.4	1.08 (0.99, 1.19)
Matched	–	–	–	98.9%/88.4%	11.98 (2.36, 60.74)	1.12 (1.02, 1.23)	8.59 (1.49, 48.52)	0.8	1.09 (0.99, 1.21)
Any TRAE Grade 3+									
Observed	51.4%/49.8%	1.06 (0.77, 1.47)	1.03 (0.88, 1.21)	46.7%/46.4%	1.01 (0.61, 1.67)	1.01 (0.77, 1.32)	0.95 (0.52,1.72)	57.0	0.98 (0.71, 1.33)
Exclusion criteria applied	–	–	–	48.5%/50.0%	0.94 (0.53, 1.67)	0.97 (0.72, 1.30)	0.89 (0.46, 1.71)	64.0	0.94 (0.67, 1.31)
Matched	–	–	–	45.4%/51.5%	0.78 (0.38, 1.59)	0.88 (0.61, 1.28)	0.74 (0.34, 1.60)	77.9	0.86 (0.57, 1.28)
Any TEAE									
Observed	98.0%/99.0%	0.50 (0.12, 2.03)	0.99 (0.97, 1.01)	98.5%/97.3%	1.83 (0.30, 11.15)	1.01 (0.98, 1.05)	3.63 (0.37, 35.55)	13.4	1.02 (0.98, 1.07)
Exclusion criteria applied	–	–	–	98.1%/96.5%	1.83 (0.30, 11.18)	1.02 (0.97, 1.07)	3.62 (0.37, 35.55)	13.5	1.03 (0.97, 1.08)
Matched	–	–	–	98.9%/97.9%	1.93 (0.30, 13.61)	1.01 (0.98, 1.04)	3.83 (0.37, 39.69)	13.0	1.02 (0.98, 1.06)
Any serious TEAE									
Observed	46.6%/44.0%	1.11 (0.80, 1.54)	1.06 (0.89, 1.27)	41.5%/42.0%	0.98 (0.59, 1.63)	0.99 (0.73, 1.33)	0.88 (0.48, 1.61)	66.0	0.93 (0.66, 1.32)
Exclusion criteria applied	–	–	–	38.8%/40.7%	0.93 (0.52, 1.66)	0.95 (0.67, 1.36)	0.83 (0.43, 1.62)	70.7	0.90 (0.60, 1.34)
Matched	–	–	–	45.0%/38.9%	1.29 (0.62, 2.66)	1.16 (0.75, 1.78)	1.16 (0.52, 2.55)	36.0	1.09 (0.68, 1.73)
TEAE leading to treatment withdrawal									
Observed	17.2%/17.5%	0.98 (0.64, 1.50)	0.98 (0.69, 1.40)	14.1%/17.9%	0.75 (0.38, 1.49)	0.79 (0.44, 1.41)	0.77 (0.34, 1.72)	74.1	0.80 (0.41, 1.58)
Exclusion criteria applied	–	–	–	14.6%/15.1%	0.96 (0.43, 2.14)	0.96 (0.48, 1.92)	0.98 (0.39, 2.42)	52.1	0.98 (0.45, 2.12)
Matched	–	–	–	16.2%/17.3%	0.93 (0.34, 2.51)	0.94 (0.40, 2.18)	0.94 (0.32, 2.78)	54.2	0.95 (0.38, 2.37)
TEAE leading to death									
Observed	7.1%/5.5%	1.31 (0.67, 2.57)	1.29 (0.69, 2.43)	4.4%/6.3%	0.70 (0.23, 2.14)	0.71 (0.24, 2.07)	0.53 (0.14, 1.95)	83.0	0.55 (0.16, 1.90)
Exclusion criteria applied	–	–	–	3.9%/5.8%	0.65 (0.17, 2.52)	0.67 (0.18, 2.43)	0.50 (0.11, 2.24)	81.8	0.52 (0.12, 2.17)
Matched	–	–	–	7.4%/5.2%	1.46 (0.33, 6.52)	1.43 (0.35, 5.90)	1.11 (0.21, 5.69)	44.9	1.10 (0.23, 5.19)
Any TRAE Grade 3+									
Observed	70.9%/66.3%	1.24 (0.87, 1.76)	1.07 (0.96, 1.19)	63.7%/64.3%	0.98 (0.58, 1.64)	0.99 (0.82, 1.20)	0.79 (0.42, 1.47)	77.6	0.93 (0.74, 1.15)
Exclusion criteria applied	–	–	–	64.1%/64.0%	1.01 (0.55, 1.83)	1.00 (0.81, 1.24)	0.81 (0.41, 1.61)	72.7	0.94 (0.73, 1.19)
Matched	–	–	–	66.7%/62.1%	1.22 (0.59, 2.55)	1.07 (0.82, 1.40)	0.98 (0.43, 2.22)	51.5	1.00 (0.75, 1.34)

Abbreviations: CI, confidence interval; CrI, credible interval; EV, enfortumab vedotin; OR, odds ratio; RR, risk ratio; TEAE, treatment emergent adverse event; TRAE, treatment related adverse event.

## DISCUSSION

To our knowledge, this study is the first to explore the relative efficacy and safety of erdafitinib vs EV in patients with la/mUC whose disease progressed after 1 or 2 prior treatments, at least 1 of which included an anti-PD(L)-1 agent. An anchored MAIC was conducted to align with EV-301’s eligibility criteria (ECOG PS <2, patients with 1 prior platinum-based chemotherapy) and to adjust for differences in the measured baseline characteristics between patients in the THOR (Cohort 1) vs the EV-301 trial. Additionally, to ensure an equitable comparison between erdafitinib and EV for cORR and cCR, the analyses were based on response-evaluable populations from THOR to match the approach utilized in EV-301. Population matching resulted in a decrease in ESS from 197 to 126 patients (a 36% reduction), which was considered sufficient to support comparisons for efficacy and safety outcomes. Given that the ITT populations were well-balanced after matching, it was anticipated that the response-evaluable subgroups of similarly selected patients in both trials were alike, thereby minimizing any impact on the comparison results.

In the base-case analysis, erdafitinib indicated a small numeric advantage over EV for survival, with a probability of being better than EV of approximately 62.1% for OS and 60.5% for PFS. In terms of cORR, erdafitinib had a 72.6% probability of being better than EV. The advantage was more pronounced in terms of cCR, where erdafitinib had an 81.3% probability of being better than EV. In the current study, the findings were consistent across all sensitivity analyses that considered different baseline characteristics for adjustment, lines of prior therapy, and the available 23.75-month median follow-up data for EV-301, supporting the robustness of the comparative efficacy results.

With respect to the safety analyses, patients treated with erdafitinib were more likely to experience any TRAEs than EV-treated patients but less likely to experience high-grade TRAEs compared with EV. In addition, erdafitinib was associated with a slightly higher occurrence of any TEAEs, but for the remaining TEAEs, including serious or grade 3+ TEAEs and TEAEs leading to withdrawal or death, outcomes were similar between the two drugs. Of note, when comparing the safety of erdafitinib and EV in terms of any TRAE or any TEAE, the RRs were near 1 while the ORs point-estimates were greater than 1. This difference relates to the high rates of AEs in both trials (>90%), as the OR is very sensitive to boundary cases (proportions near 0 or 1). For all other safety measures (ie, any grade 3+ TRAE, any grade 3+ AE, any serious AE, AE leading to treatment withdrawal, AE leading to death), the probability of erdafitinib being better than EV ranged from 36.0% to 77.9% and all 95% CrIs included the null effect.

This study has a few limitations. A systematic identification of treatment effect modifiers in UC was not carried out for this MAIC; however, the adopted approach allowed all baseline characteristics that were mutually reported in THOR and EV-301 to be eligible for adjustment. In addition, cumulative adjustment for each baseline characteristic on a one-by-one basis in order of clinical significance as determined by physician expert consultation produced consistent results with sufficient ESS; therefore, any risk of overadjustment was considered negligible. Nevertheless, possible bias due to unmeasured or unknown treatment effect modifiers is an inherent limitation of any MAIC that we cannot rule out. There were some known patient characteristics that could not be adjusted for including presence of lymph node-only metastasis, best response to ICI, and median time since diagnosis of metastatic or locally advanced disease, since they were not recorded similarly in THOR and EV-301.

In addition, it was not possible to adjust for differences in the distribution of patients with FGFR alterations since THOR exclusively recruited patients with FGFR alterations and EV-301 did not assess FGFR status. THOR only recruited patients with 2 or fewer prior systemic therapies, whereas a small but notable proportion of EV-301 patients had at least 3 prior systemic therapies, making it impossible to completely adjust for differences in this baseline characteristic. Nevertheless, sensitivity analyses using EV-301 subgroup data for patients with 2 or fewer prior systemic therapies were generally similar to the base-case MAIC OS and PFS results. Of note, the MAIC PFS results are considered conservative for erdafitinib, as more frequent radiographic assessments in THOR compared with EV-301 (every 6 weeks vs every 8 weeks) may have allowed earlier detection of progression. The impact of the different measurement timeframes on ORR and CR is less clear; however, any bias is expected to be minimal.

With respect to median follow-up time, THOR (15.9 months) was slightly longer than the EV-301 interim data (11.1 months) but shorter than the longer-term follow-up data (23.75 months). Because rates of observed AEs may be higher over a longer follow-up time, a sensitivity analysis was conducted using data from the longer follow-up time in EV-30116; however, TEAEs were not reported for this analysis. The relative treatment effects between trials were assumed consistent over time. Ultimately, the relative efficacy and safety of erdafitinib vs EV (23.75 months) was similar to that seen in the base case. In addition, we observed evidence of a minor deviation from the proportional hazards assumption for the comparison of erdafitinib vs chemotherapy in terms of OS and PFS, both before and after matching. Thus, the HR of erdafitinib vs chemotherapy represents a summary of HRs that might slightly vary over time. Consequently, the relative efficacy of erdafitinib vs EV could also display variability across different time periods, though this variation is expected to be very limited. Finally, as the MAIC is a post hoc analysis of trials, it may not be statistically powered to detect a difference in treatment effect with high certainty. The ESS was limited by the original size of the trials and was further reduced by matching the trial populations, which increased the size of the CrIs.

Novel treatment options offering meaningful survival benefits for patients with la/mUC that has progressed following chemotherapy and/or ICI treatment are limited. Targeted therapy and antibody drug conjugates are important recent additions to the therapeutic armamentarium.^26^ According to current National Comprehensive Cancer Network guidelines for second-line treatment of la/mUC, EV and erdafitinib (for patients with FGFR alterations) are considered “alternative preferred regimens” in the postchemotherapy setting, and preferred regimens in the post-ICI setting in cisplatin ineligible, chemotherapy-naïve patients.^5^ Erdafitinib is also a preferred second-line regimen for cisplatin eligible, chemotherapy-naïve patients who previously received an ICI.^5^ European Society for Medical Oncology guidelines recommend erdafitinib for patients with la/mUC containing FGFR alterations whose disease has progressed following EV and pembrolizumab combination therapy, cisplatin-based chemotherapy or carboplatin-based therapy with avelumab maintenance or followed by an ICI or after progression on a cisplatin-based therapy in combination with nivolumab.^6^ Similarly, guidelines from the European Association of Urology endorse erdafitinib for patients with la/mUC containing FGFR alterations whose disease has progressed following EV and pembrolizumab combination therapy or alternatively platinum-containing chemotherapy with maintenance avelumab or in combination with nivolumab, in cases of eligibility for an ICI, or after progression on a single agent ICI in cases of noneligibility to the EV and pembrolizumab combination.^7^ Other than its recommended use in combination with pembrolizumab in the first-line setting for eligible patients, EV is recommended as treatment after chemotherapy among cisplatin-eligible patients and postchemotherapy with avelumab maintenance or post-ICI among cisplatin-ineligible patients.^7^

While FGFR status was not explicitly addressed in this analysis, the results support erdafitinib as an effective treatment option for FGFR3+ patients with la/mUC patients whose disease has progressed after at least 1 line of therapy containing a PD-1 or PD(L)-1 inhibitor.

## CONCLUSION

The MAIC indicates comparable efficacy of erdafitinib vs EV for OS and PFS, with erdafitinib showing a higher probability of achieving deep responses. While erdafitinib is associated with slightly more AE than EV, these events seem to be less severe.

### Disclosures

S.V. is an employee of Janssen and owns stock in Johnson & Johnson. A.Y. is an employee of Janssen/Johnson & Johnson. S.B. is an employee of Janssen and owns stock in Johnson & Johnson. K.S. is an employee of Janssen and owns stock in Johnson & Johnson. S.T. is an employee of Janssen and owns stock in Johnson & Johnson. Z.Y. is an employee of Cytel, which was contracted for this work by Janssen Pharmaceutica NV. C.D. was an employee of Cytel at the time of writing, which was contracted for this work by Janssen Pharmaceutica NV.

## Supplementary Material

Online Supplementary Material
