# Supplementary material for: Matching-Adjusted Indirect Comparison of the Efficacy and Safety of Erdafitinib vs Enfortumab Vedotin in Patients with Locally Advanced Metastatic Urothelial Carcinoma
Source: J Health Econ Outcomes Res. 2024 Aug 20;11(2):49–57. doi: 10.36469/001c.120954 (PMC11392482; doi:10.36469/001c.120954)
Supplement: Online Supplementary Material [file jheor_2024_11_2_120954_242503.pdf]

Journal of Health Economics and Outcomes Research

Online Supplementary Material

Matching-Adjusted Indirect Comparison of the Efficacy and Safety of Erdafitinib vs Enfortumab Vedotin in Patients With Locally Advanced Metastatic Urothelial Carcinoma. *JHEOR*. 2024;11(2):49-57. doi:10.36469/jheor.2024.120954

Table S1: Study Characteristics of THOR and EV-301

Table S2: Key Inclusion and Exclusion Criteria in THOR and EV-301

Table S3: Treatment Characteristics in THOR and EV-301

Table S4: Endpoint Definitions in THOR and EV-301

This supplementary material has been provided by the authors to give readers additional information about their work.

This is an open-access article distributed under the terms of the Creative Commons Attribution 4.0 International License (CCBY-4.0). View this license's legal deed at http://creativecommons.org/licenses/by/4.0 and legal code at http://creativecommons.org/licenses/by/4.0/legalcode for more information.

Table S1. Study Characteristics of THOR and EV-301

	THOR	EV-301
Study design	RCT	RCT
Trial phase	Phase 3	Phase 3
Blinding	Open label	Open label
Concealment of randomization	Adequate	Adequate
Strata during randomization	• Geographic region (North America vs EU vs rest of the world)<br>• ECOG PS ([0 or 1] vs 2)<br>• Presence of visceral metastasis: lung, liver, or bone (yes vs no)	• Geographic region (Western Europe vs US vs rest of the world)<br>• ECOG PS (0 vs 1)<br>• Presence of liver metastasis (yes vs no)
No. of patients<br>(ITT population)	Cohort 1:<br>Erdafitinib: 136<br>Physician's choice of chemotherapy: 130	EV: 301<br>Physician's choice of chemotherapy: 307
Location	International	International
Median follow-up	Cohort 1:<br>Interim/final*: 15.9 months	Interim analysis: 11.1 months<br>Longer-term analysis: 23.75 months
Cross-over	Crossover allowed after interim analysis$	Allowed if positive results observed in interim analysis$

* Cohort 1 was stopped at interim analysis due to superiority of erdafitinib over chemotherapy; interim analysis is considered final analysis.

$ In both trials, there is no cross-over within the interim analysis data-cut, which served as the basis for the MAIC. However, it remains uncertain whether the longer-term analysis for EV-301 include any instances of cross-over, and if so, whether the analysis has been appropriately adjusted to account for it.

Abbreviations: ECOG PS, Eastern Cooperative Oncology Group performance status; EU, European Union; EV, enfortumab vedotin; ITT, Intention-to-treat; OS, overall survival; PFS, progression-free survival; RCT, randomized controlled trial; US, United States.

Table S2. Key Inclusion and Exclusion Criteria in THOR and EV-301

	THOR (NCT03390504)	EV-301 (NCT03474107)
**Inclusion criteria**		
Age	≥18 years	≥ 18 years
Disease status	• Histologic demonstration of transitional cell carcinoma of the urothelium<br>• Metastatic or surgically unresectable urothelial cancer	• Histologically or cytologically confirmed urothelial carcinoma<br>• Has radiologically documented metastatic or locally advanced disease at baseline
Progression and prior treatment	• Documented progression of disease, defined as any progression that requires a change in treatment, prior to randomization<br>• Prior treatment with an anti-PD(L) 1 agent as monotherapy or as combination therapy, given as neo-adjuvant, adjuvant, or in metastatic line of treatment as frontline or maintenance therapy<br>• ≤2 prior lines of systemic treatment	• Have experienced radiographic progression or relapse during, or after CPI (anti-PD-1 or anti-PD-L1) for locally advanced or metastatic disease<br>• Have received a platinum containing regimen in the metastatic/locally advanced, neoadjuvant or adjuvant setting. If platinum was administered in the adjuvant/neoadjuvant setting subject must have progressed within 12 months of completion.
ECOG PS	0-2	0-1
Baseline laboratory data	• ANC ≥1500/mm^3<br>• Platelet count >75,000/mm^3 (≥100,000/mm^3 for Cohort 1 subjects at sites choosing vinflunine chemotherapy)<br>• Hemoglobin >8.0 g/dL<br>• Total bilirubin ≤1.5 x ULN OR direct bilirubin ≤ ULN for subjects with total bilirubin levels >1.5xULN [≤1xULN for Cohort 1 subjects at sites choosing docetaxel chemotherapy]<br>• CrCl >30 mL/min either directly measured via 24-hour urine collection or calculated using the Cockcroft-Gault formula<br>• ALT and AST ≤2.5x institutional ULN or ≤5x institutional ULN for subjects with liver metastases (For subjects in Cohort 1 at sites choosing docetaxel chemotherapy, both the ALT and AST values must be ≤1.5×ULN concomitant with alkaline phosphatase of ≤2.5×ULN)<br>• Phosphate: <ULN within 14 days of treatment and prior to Cycle 1 Day 1 (medical management allowed)	• ANC ≥1500/mm^3<br>• Platelet count ≥100 x 10^9/L<br>• Hemoglobin ≥9 g/dL<br>• Serum total bilirubin ≤1.5x ULN or ≤3x ULN for subjects with Gilbert's disease<br>• CrCl ≥30 mL/min as estimated per institutional standards or as measured by 24hour urine collection (GFR can also be used instead of CrCl)<br>• ALT and AST ≤2.5 × ULN or ≤3x ULN for subjects with liver metastases
Molecular	• Tumors must have ≥1 of the following translocations: FGFR2-BICC1, FGFR2-CASP7, FGFR3- TACC3, FGFR3-BAIAP2L1; or 1 of the following FGFR3 gene mutations: R248C, S249C, G370C, Y373C	
**Exclusion criteria**		

	THOR<br>(NCT03390504)	EV-301<br>(NCT03474107)
Disease status	• Active malignancies (requiring treatment change in the last 24 months) with the exception of<br>o urothelial cancer<br>o Skin cancer treated within the last 24 months that is considered completely cured<br>o localized prostate cancer with a Gleason score of 6 (treated within the last 24 months or untreated and under surveillance)<br>o localized prostate cancer with a Gleason score of 3+4 that has been treated more than 6 months prior to full study screening and considered to have a very low risk of recurrence<br>• Symptomatic CNS metastases<br>• Current CSR or retinal pigment epithelial detachment of any grade	• Has preexisting sensory or motor neuropathy Grade ≥2<br>• Has active CNS metastases
Prior treatment	• Received prior FGFR inhibitor treatment<br>• Not recovered from reversible toxicity of prior anticancer therapy<br>• Major surgery within 4 weeks before randomization	• Prior treatment with EV or other MMAE-based ADCs<br>• Received prior chemotherapy for UC with all available study therapies in the control arm<br>• Received >1 prior chemotherapy regimen for locally advanced or metastatic urothelial cancer<br>• Ongoing clinically significant toxicity (≥ Grade 2 with the exception of alopecia) associated with prior treatment<br>• Radiotherapy or major surgery within 4 weeks prior to first dose of study drug
Medical history	• History of uncontrolled cardiovascular disease<br>• Known active AIDS (human immunodeficiency virus (HIV) infection)<br>• Known active hepatitis B or C infection<br>• Impaired wound healing capacity defined as skin/decubitus ulcers, chronic leg ulcers, known gastric ulcers, or unhealed incisions	• History of another malignancy within 3 years<br>• History of a cerebral vascular, unstable angina, myocardial infarction, or cardiac symptoms (NYHAC III-IV) within 6 months prior trial<br>• Known history of HIV infection<br>• Known active hepatitis B or C<br>• Known active keratitis or corneal ulcerations<br>• History of uncontrolled diabetes mellitus within 3 months prior study

Abbreviations: ADC, antibody drug conjugate; AIDS, acquired immune deficiency syndrome; ALT, alanine aminotransferase; ANC, absolute neutrophil count; AST, aspartate aminotransferase; CNS, central nervous system; CPI, checkpoint inhibitor; CrCl, Creatinine Clearance; CSR, central serous retinopathy; EV, enfortumab vedotin; FGFR, fibroblast growth factor receptor; GFR, glomerular filtration rate; HIV, human immunodeficiency virus; NYHAC, New York Heart Association (NYHA) Classification; MMAE, monomethyl auristatin E; PD-(L)1, programmed cell death protein/programmed cell death ligand-1; ULN, upper limit of normal

Table S3. Treatment Characteristics in THOR and EV-301

	THOR		EV-301	
Intervention	Erdafitinib	Physician's choice of chemotherapy<br>(Vin, Doc)	EV	Physician's choice of chemotherapy<br>(Vin, Doc, Pac)
Dose	8 mg with a pharmacodynamically guided increase in the dose to 9 mg on day 14	Vin: 320 mg/m^2<br>Doc: 75 mg/m^2	1.25 mg/kg	Vin: 320 mg/m^2<br>Doc: 75 mg/m^2<br>Pac: 175 mg/m^2
Frequency and cycle length	Once daily for 21 days in a 21-day cycle	Vin/Doc: once every 3 weeks	Days 1, 8, and 15 of a 28-day cycle.	Vin/Doc/Pac: Day 1 of a 21-day cycle
Route of administration	Oral	Vin/Doc: IV	IV	Vin/Doc/Pac: IV
Treatment duration	until the occurrence of disease progression or unacceptable toxic effects.		Until radiological disease progression as determined per investigator assessment or other discontinuation criteria were met or upon study termination, or study completion, whichever occurred first.	

Abbreviations: Doc, docetaxel; EV, enfortumab vedotin; IV, intravenous; NR, not reported; Pac, paclitaxel; Vin, vinflunine.

Table S3. Endpoint Definitions in THOR and EV-301

	THOR	EV-301
**OS**		
Outcome definition	Time from the date of randomization until the documented date of death	Time from the date of randomization until the documented date of death from any cause
Time frame (median, month)	15.9	11.1*
**PFS**		
Criteria	RECIST v1.1	RECIST v1.1
Assessor	Investigator	Investigator
Outcome definition	Time from the date of randomization to the date of disease progression or relapse from complete response or death, whichever is reported first divided	Time from date of randomization until date of documented radiological disease progression or until death due to any cause, whichever occurred first
Timing of assessment	Every 6 weeks (± 7 days)^	Every 8 weeks (± 7 days)
Time frame (median, month)	15.9	11.1*
**cORR**		
Criteria	RECIST v1.1	RECIST v1.1
Assessor	Investigator	Investigator
Outcome definition	Proportion of participants who achieve CR or PR	Proportion of participants with CR or PR
Timing of assessment	Every 6 weeks (± 7 days)^	Every 8 weeks (± 7 days)
Time frame (median, month)	15.9	11.1
**cCR**		
Criteria	RECIST v1.1	RECIST v1.1
Assessor (BICR, investigator, other)	Investigator	Investigator
Outcome definition	NR	Disappearance of all target and nontarget lesions
Timing of assessment	Every 6 weeks (± 7 days)^	Every 8 weeks (± 7 days)
Time frame (median, month)	15.9	11.1
**Adverse events**		
Criteria	CTCAE v 4.03	CTCAE v 4.03
Assessor	Subject	Investigator
Time frame	From date of signed informed consent up to 30 days after last dose	From date of signed informed consent up to 30 days after last dose

*A longer follow-up time of month 23.75 is available for OS, PFS, and TRAEs.
^Every 6 weeks (± 7 days) for the first year, and then as per clinically indicated.
Abbreviations: BICR, Blinded Independent Central Review; cCR, confirmed complete response; cORR, confirmed objective or overall response rate; CTCAE, National Cancer Institute Common Terminology Criteria for Adverse Events; NR, not reported; PR, partial response.

Table S5. Patient Demographics and Baseline Characteristics in THOR and EV-301

	THOR NCT03390504		EV-301 NCT03474107	
	Erdafitinib (N, 136)	Chemotherapy (N, 130)	EV (N, 301)	Chemotherapy (N, 307)
**Age in years**				
Median (min, max)	66.0 (32, 85)	69.0 (35, 86)	68.0 (34.0, 85.0)	68.0 (30.0, 88.0)
≥75, n (%)	26 (19.1)	30 (23.1)	52 (17.3)	68 (22.1)
**Sex, n (%)**				
Male	96 (70.6)	94 (72.3)	238 (79.1)	232 (75.6)
**Geographic region n (%)**				
Europe	82 (60.3)	80 (61.5)	126 (41.9)	129 (42.0)
North America	8 (5.9)	5 (3.8)	43 (14.3)	44 (14.3)
Rest of the world	46 (33.8)	45 (34.6)	132 (43.9)	134 (43.6)
**Race, n (%)**				
White	81 (59.6)	63 (48.5)	–	–
Asian	37 (27.2)	40 (30.8)	–	–
Black or African American	0	1 (0.8)	–	–
Multiple	0	1 (0.8)	–	–
Not reported	18 (13.2)	25 (19.2)	–	–
**Tobacco use, n (%)**				
Former user	–	–	167 (55.5)	164 (53.4)
Current user	–	–	29 (9.6)	31 (10.1)
Never used	44 (32.4)	47 (36.2)	91 (30.2)	102 (33.2)
Not reported or unknown	–	–	14 (4.7)	10 (3.3)
**History of diabetes or hyperglycemia, n (%)**				
Yes	11 (8.1)	22 (16.9)	56 (18.6)	58 (18.9)
**ECOG PS score, n (%)**				
0	63 (46.3)	51 (39.2)	120 (39.9)	124 (40.4)
1	61 (44.9)	66 (50.8)	181 (60.1)	183 (59.6)
2	12 (8.8)	13 (10.0)	-	-
**Bellmunt risk score, n (%)**				
0–1	103 (75.7)	95 (73.1)	201 (66.8)	208 (67.8)
≥2	33 (24.3)	35 (26.9)	90 (29.9)	96 (31.3)
Not reported	–	–	10 (3.3)	3 (1.0)
**Origin site of primary disease, n (%)**				
Upper urinary tract	41 (30.1)	48 (36.9)	98 (32.6)	107 (34.9)
Bladder or other site	95 (69.9)	82 (63.1)	203 (67.4)	200 (65.1)
**Sites of metastasis, n/total (%)**				
Lymph node only	–	-	34/301 (11.3)	28/306 (9.2)
Visceral site	101 (74.3)	97 (74.6)	234/301 (77.7)	250/306 (81.7)

	THOR NCT03390504		EV-301 NCT03474107	
	Erdafitinib (N, 136)	Chemotherapy (N, 130)	EV (N, 301)	Chemotherapy (N, 307)
Liver	31 (22.8)	38 (29.2)	93/301 (30.9)	95/307 (30.9)
**PD(L)-1 status**				
Low expression (CPS <10), n (%)	89 (92.7)	68 (86.1)	–	-
**FGFRa/t, n/total (%)**				
Mutations	108/135 (79.4)	107/129 (82.3)	–	-
Fusions	25/135 (18.4)	19/129 (14.6)	–	-
Mutations and fusions	2/135 (1.5)	3/129 (2.3)	–	-
**Histologic type at initial diagnosis, n/total (%)**				
Urothelial or transitional-cell carcinoma	–	–	229/301 (76.1)	230/305 (75.4)
Urothelial carcinoma, mixed types	–	–	45/301 (15.0)	42/305 (13.8)
Other§	–	–	27/301 (9.0)	33/305 (10.8)
**Histologic type at baseline, n (%)**				
Transitional cell carcinoma	128 (94.1)	124 (95.4)		
Transitional cell carcinoma with minor components (<50% overall) of variant histology	8 (5.9)	6 (4.6)		
**Previous systemic therapies, n (%)**				
1–2	135 (99.3)	130 (100)	262 (87.0)	270 (87.9)
≥3	1 (0.7)	–	39 (13.0)	37 (12.1)
**Prior platinum-based chemotherapy, n (%)**				
None	14 (10.3)	19 (14.6)	0	0
**Best response among patients who previously received checkpoint inhibitor treatment, n (%)¶**				
Response	–	–	61 (20.3)	50 (16.3)
No response	–	–	207 (68.8)	215 (70.0)
**Time since diagnosis of metastatic or locally advanced disease in months**				
Median (min, max)	–	–	14.8 (0.2, 114.1)	13.2 (0.3, 118.4)
**Time from diagnosis of surgically unresectable or metastatic disease to randomization in months**				
Median (min, max)	12.9 (0.6, 74.6)	11.7 (1.8, 63.5)	–	–

* Percentages may not total 100 because of rounding.
§ Other histologic types include adenocarcinoma, squamous-cell carcinoma, and pseudosarcomatic differentiation.
The best response among patients who had a response was defined as a confirmed complete or partial response; among patients who did not have a response, the best response was defined as stable disease or progressive disease.
Abbreviations: CPS, combined positive score; ECOG PS, Eastern Cooperative Oncology Group performance status; FGFR, fibroblast growth factor receptor; Max, maximum; Min, minimum; PD(L)-1, programmed cell death protein/programmed cell death ligand-1.

Table S6. Baseline Characteristics Available for Trial Population Comparison, Prioritized by Clinical Relevance According to Clinical Experts

Rank*	Characteristic
1	Bellmunt risk score (0-1, ≥2)
2	ECOG PS (0, 1)
3	Presence of liver metastases (yes, no)
4	Presence of visceral metastases (yes, no)
5	Origin of primary disease (upper urinary tract, bladder or other site)
6	Smoking status (never smoked**, other)
7	History of diabetes or hyperglycemia (yes, no)
8	Geographic region (Western Europe, US, rest of the world)
9	Age (median; ≥75 years, < 75 years)
10	Male (yes, no)

* Ranked from (1) being the most likely to (10) being the least likely to be a treatment effect modifier.
** “Other” includes “former user,” “current user,” “not reported or unknown.”
Abbreviations: ECOG PS, Eastern Cooperative Oncology Group performance status.

Table S7. Baseline characteristics reported in EV-301 that could not be appropriately matched

Characteristic	Reason for exclusion
Histologic type at initial diagnosis (urothelial or transitional-cell carcinoma, urothelial carcinoma mixed types, other)	Categories not comparable to those recorded in THOR
Presence of lymph node only metastases (yes, no)	Presence of lymph node metastases was recorded in THOR, but data does not indicate if it was the only type of metastases
Prior systemic therapies (≥3)	THOR limited to 1-2 prior therapies
Best response among patients who previously received CPI (response, no response)	Not recorded in THOR
Median time since diagnosis of metastatic or locally advanced disease	THOR only recorded time since diagnosis (not time since metastatic diagnosis)

Abbreviations: CPI, checkpoint inhibitor; ITC, indirect treatment comparison.

Table S8. Sensitivity Analysis: OS and PFS Results for Erdafitinib vs 1 or 2 Prior Lines Subgroup from EV-301

	Erdafitinib vs EV	
	**Base Case**	**1-2 Prior Lines**
OS HR (95% CrI)	0.92 (0.54, 1.57)	0.93 (0.54, 1.60)
PFS HR (95% CrI)	0.93 (0.55, 1.56)	0.90 (0.54, 1.52)

Abbreviations: CrI, credible interval; EV, enfortumab vedotin; HR, hazard ratio; PFS, progression-free survival; OS, overall survival.

Table S9. Sensitivity Analysis: Erdafitinib vs EV Results with Short-term and Longer-term Follow-up from EV-301

		Base-case Erdafitinib vs EV (11.1-Month Follow-up)		Erdafitinib vs EV (23.75-Month Follow-up)	
**Survival**	**N (ESS)**	**HR (95% CrI)**	**Probability Better than EV**	**HR (95% CrI)**	**Probability Better than EV**
OS	197 (126)	0.92 (0.54, 1.57)	62.13%	0.91 (0.54; 1.53]	63.45%
PFS	197 (126)	0.93 (0.55, 1.56)	60.50%	0.91 (0.54, 1.52)	63.57%
**Response**	**N (ESS)**	**OR (95% CrI)**	**Probability better than EV**	**OR (95% CrI)**	**Probability better than EV**
cORR	197 (126)	1.43 (0.44, 4.56)	72.63%	1.44 (0.45, 4.60)	73.25%
cCR	197 (126)	2.91 (0.27, 30.57)	81.3%	2.49 (0.24, 25.28)	78.08%
**Safety**	**N (ESS)**	**OR (95% CrI)**	**Probability better than EV**	**OR (95% CrI)**	**Probability better than EV**
Any TRAE	197 (126)	8.59 (1.49, 48.52)	0.79%	9.11 (1.58, 51.26)	0.64%
Any Grade 3+ TRAE	197 (126)	0.74 (0.34, 1.60)	77.86%	0.73 (0.33, 1.58)	78.79%

Abbreviations: cCR, confirmed complete response; cORR, objective response rate; CrI, credible interval; EV, enfortumab vedotin; ESS, effective sample size; HR, hazard ratio; OR, odds ratio; OS, overall survival; PFS, progression-free survival; TRAE, treatment related adverse event.

Table S10. Additional Safety Analysis: Erdafitinib vs EV Results for Outcomes Only Reported with Longer-term Follow-up from EV-301

	Odds Ratio (95% CrI)	Probability Erdafitinib Is Better than EV	Risk Ratio (95% CrI)
Any serious TRAE	0.56 (0.21, 1.47)	88.0%	0.62 (0.27, 1.38)
Any TRAE leading to treatment withdrawal	0.55 (0.16, 1.86)	83.0%	0.60 (0.20, 1.74)
Any TRAE leading to death	0.27 (0.02, 3.70)	83.8%	0.27 (0.02, 3.64)

Abbreviations: CrI, credible interval; EV, enfortumab vedotin; TRAE, treatment-related adverse event.

Figure S1. Sensitivity Analysis: Cumulative Baseline Characteristics Adjustment OS Results

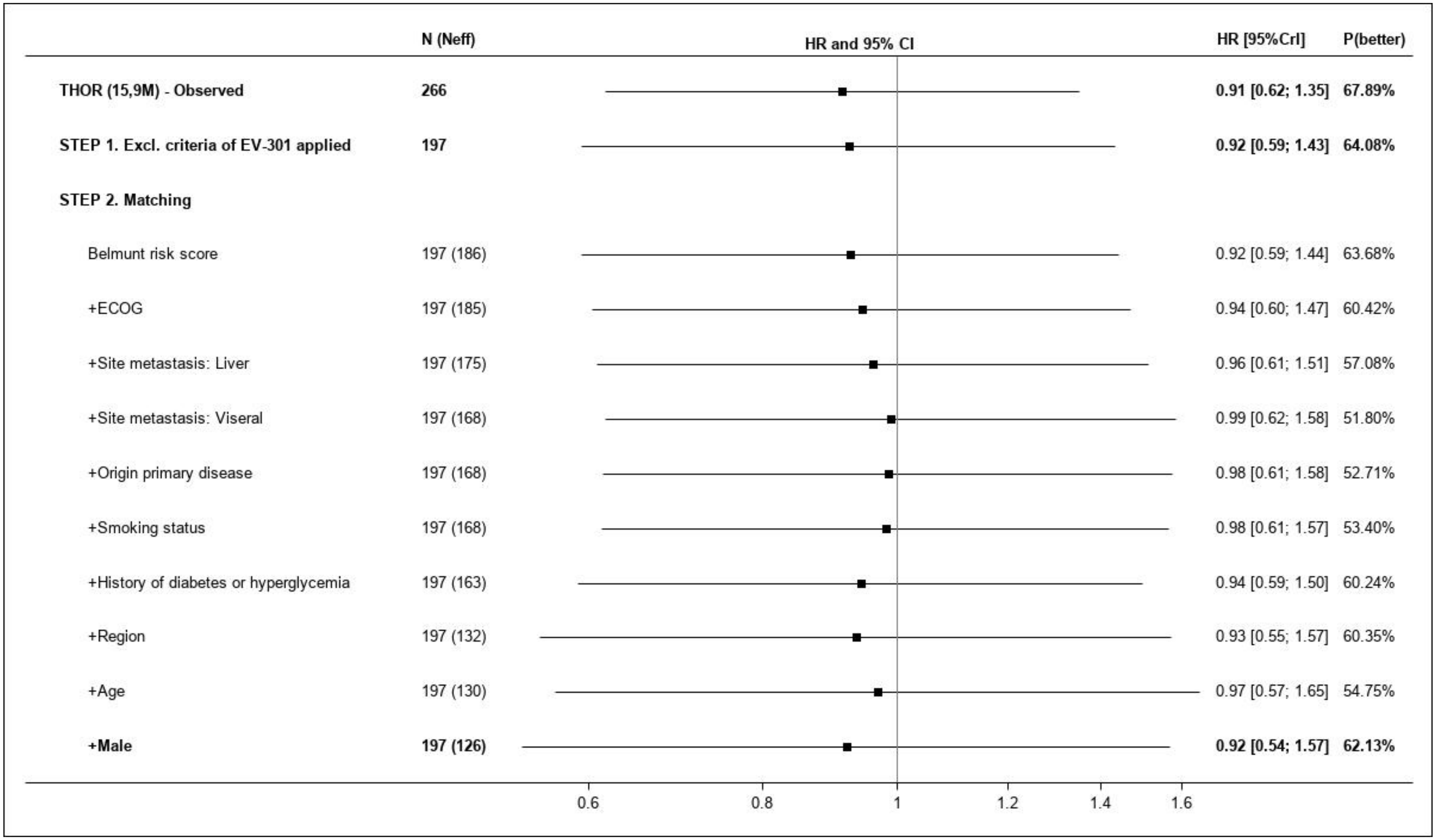

Abbreviations: CrI, credible interval; ECOG PS, Eastern Cooperative Oncology Group performance status; ESS, effective sample size; EV, enfortumab vedotin; HR, hazard ratio; Neff, effective sample size; OS, overall survival; P(better), probability of erdafitinib being better than EV.

Figure S2. Sensitivity Analysis: Cumulative Baseline Characteristic Adjustment PFS Results

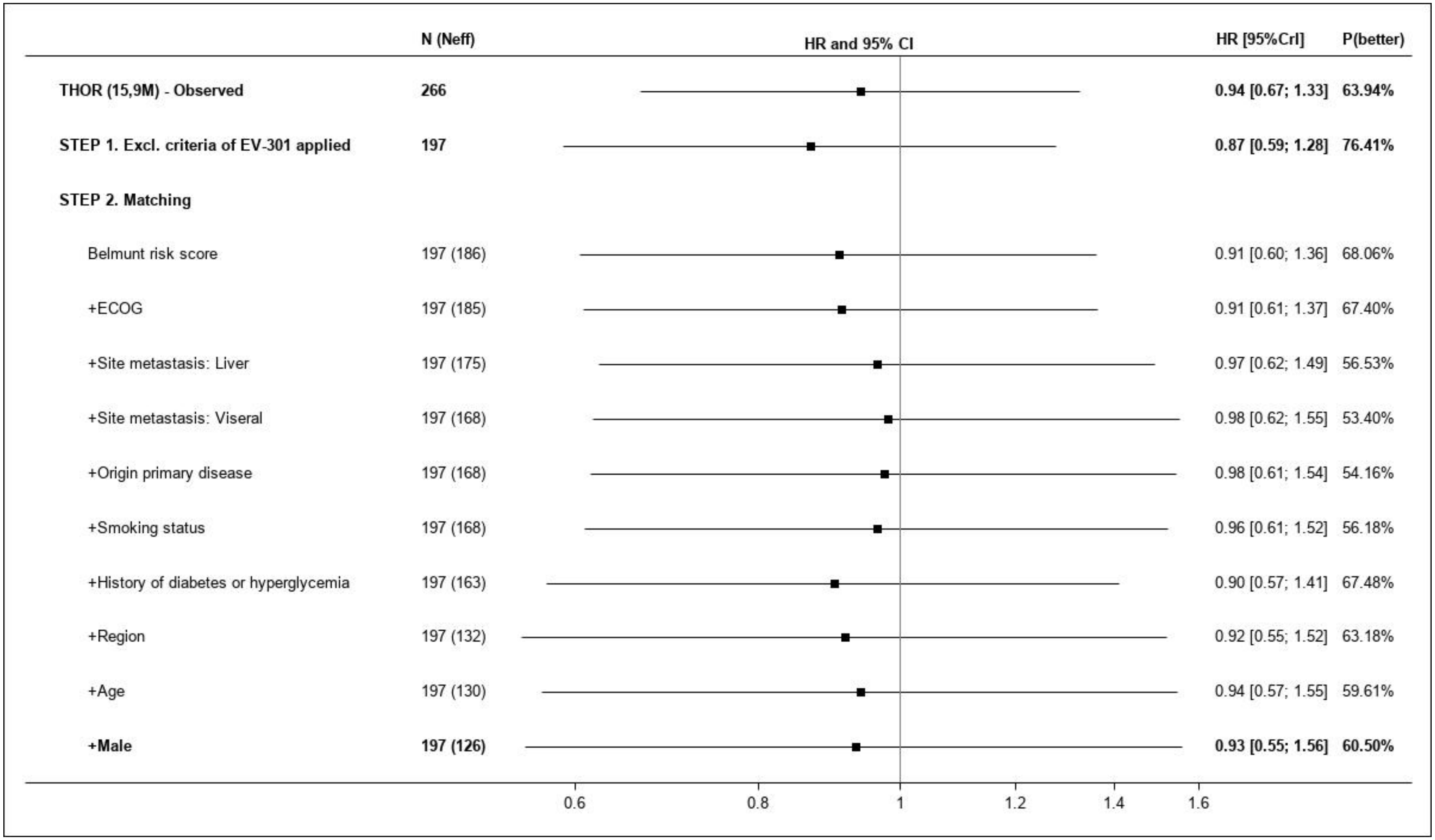

Abbreviations: CrI, credible interval; ECOG PS, Eastern Cooperative Oncology Group performance status; EV, enfortumab vedotin; HR, hazard ratio; Neff, effective sample size; PFS, progression-free survival; P(better), probability of erdafitinib being better than EV.

Figure S3. Posterior Distributions of the Hazard Ratios

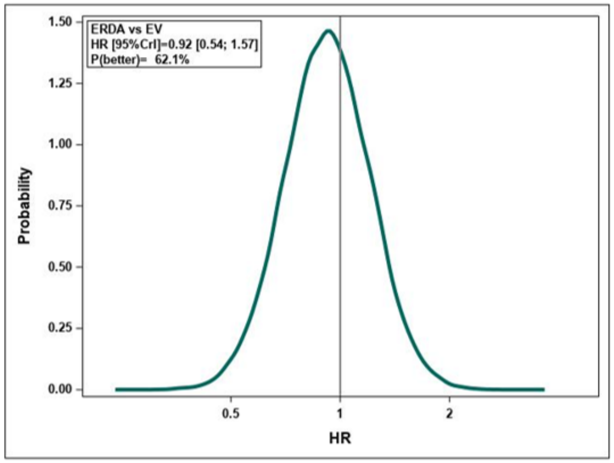

A. OS

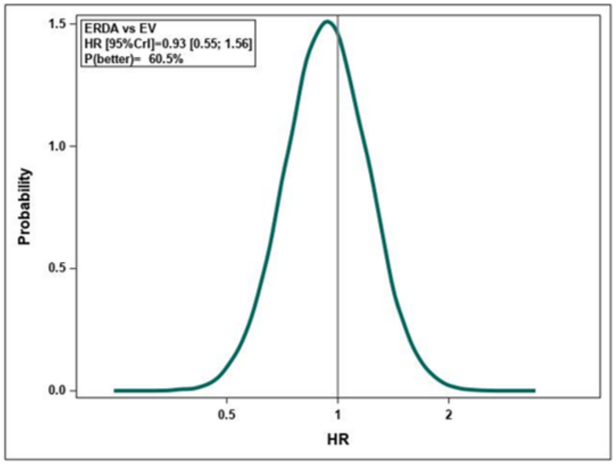

B. PFS

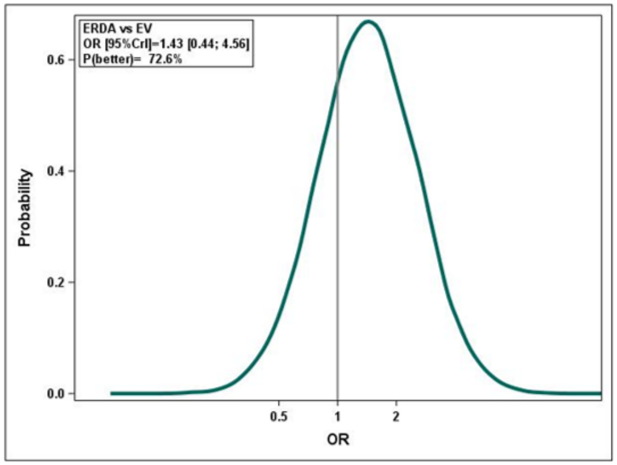

C. ORR

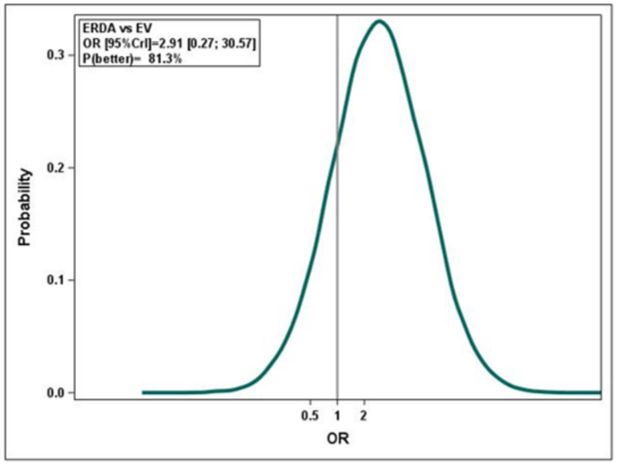

D. CR

(A) OS, (**B**) PFS, (**C**) cORR, and (**D**) cCR between erdafitinib and EV.

Abbreviations: CHEMO, chemotherapy; ERDA, erdafitinib; EV, enfortumab vedotin; HR, hazard ratio; OR, odds ratio.

Figure S4. Sensitivity Analysis: Cumulative Baseline Characteristic Adjustment cORR Results

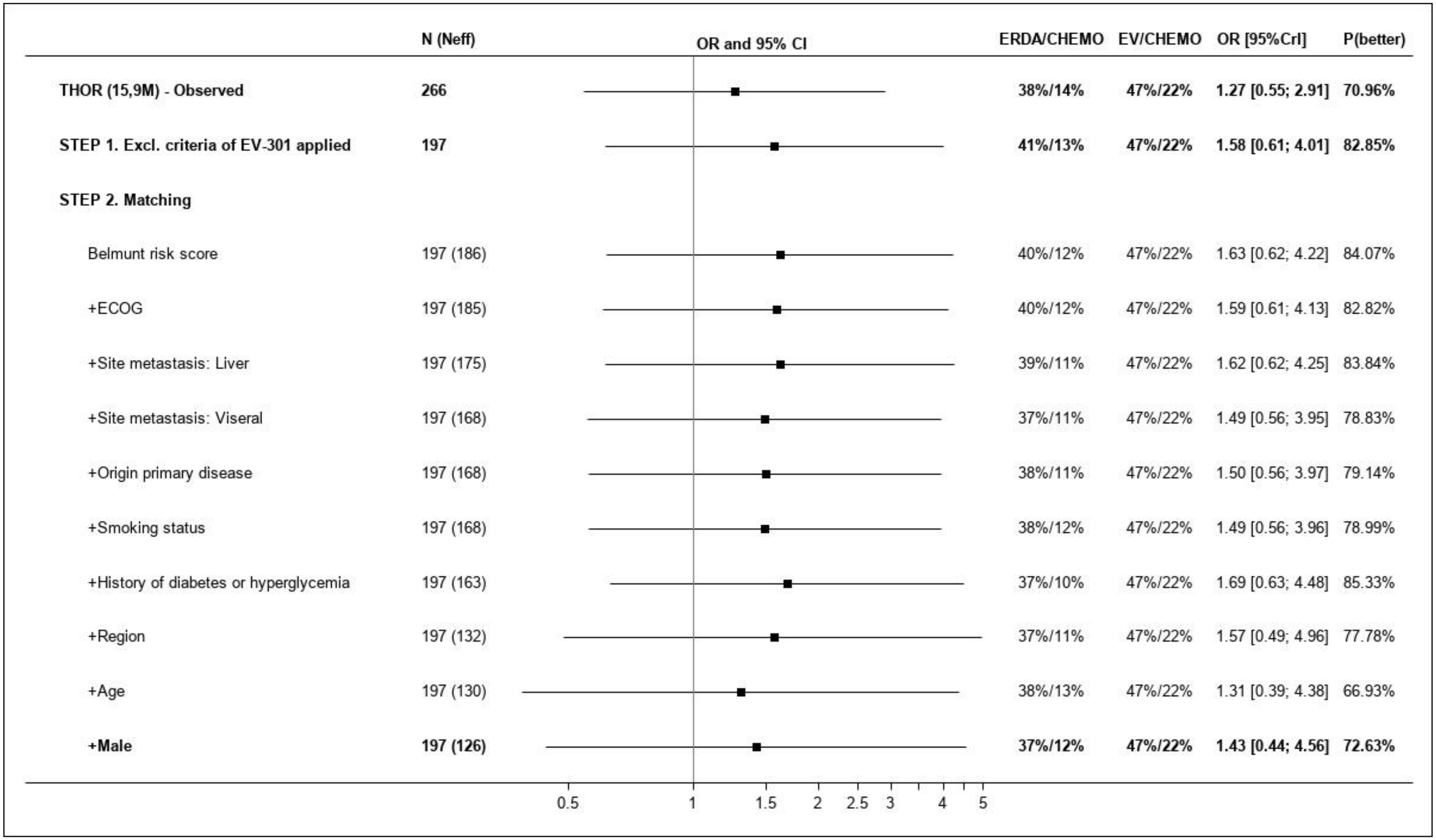

Abbreviations: cORR, confirmed objective response rate; ECOG PS, Eastern Cooperative Oncology Group performance status; ERDA, erdafitinib; ESS, effective sample size; EV, enfortumab vedotin; Neff, effective sample size; OR, odds ratio; P(better), probability of erdafitinib being better than EV.

Figure S5. Sensitivity Analysis: Cumulative Baseline Characteristic Adjustment cCR Results

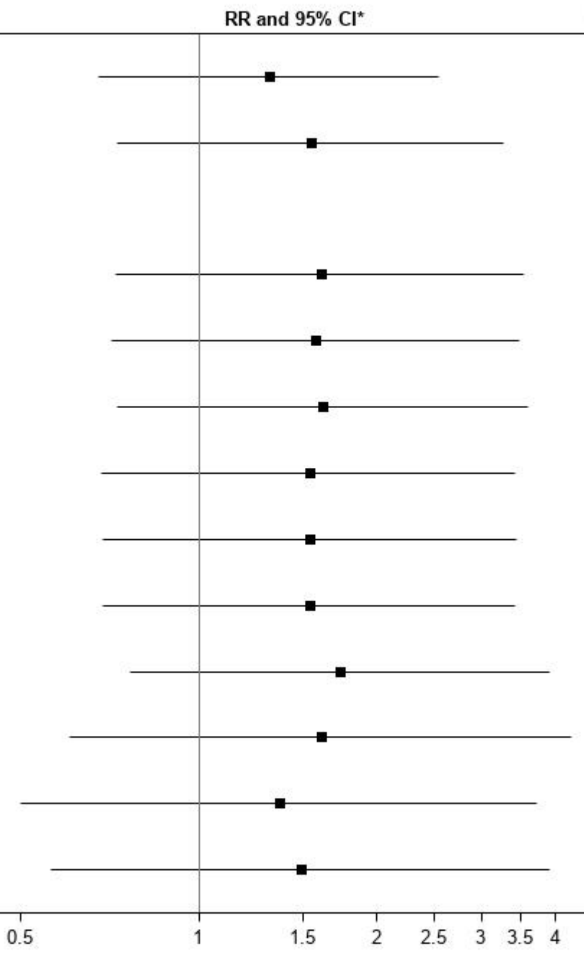

	N (Neff)	ERDA/CHEMO	EV/CHEMO	RR [95%CrI]*	P(better)*
**THOR (15,9M) - Observed**	**266**	**38%/14%**	**47%/22%**	**1.32 [0.68; 2.54]**	**79.59%**
**STEP 1. Excl. criteria of EV-301 applied**	**197**	**41%/13%**	**47%/22%**	**1.55 [0.73; 3.26]**	**87.42%**
**STEP 2. Matching**					
Belmunt risk score	197 (186)	40%/12%	47%/22%	1.61 [0.72; 3.54]	88.17%
+ECOG	197 (185)	40%/12%	47%/22%	1.58 [0.71; 3.47]	87.13%
+Site metastasis: Liver	197 (175)	39%/11%	47%/22%	1.63 [0.73; 3.59]	88.54%
+Site metastasis: Viseral	197 (168)	37%/11%	47%/22%	1.54 [0.69; 3.42]	85.36%
+Origin primary disease	197 (168)	38%/11%	47%/22%	1.54 [0.69; 3.43]	85.58%
+Smoking status	197 (168)	38%/12%	47%/22%	1.54 [0.69; 3.41]	85.46%
+History of diabetes or hyperglycemia	197 (163)	37%/10%	47%/22%	1.74 [0.77; 3.90]	90.87%
+Region	197 (132)	37%/11%	47%/22%	1.61 [0.60; 4.25]	83.28%
+Age	197 (130)	38%/13%	47%/22%	1.37 [0.50; 3.72]	73.19%
**+Male**	**197 (126)**	**37%/12%**	**47%/22%**	**1.49 [0.56; 3.90]**	**79.08%**

Abbreviations: cCR, confirmed complete response; CrI, credible interval; ECOG PS, Eastern Cooperative Oncology Group performance status; ERDA, erdafitinib; EV, enfortumab vedotin; HR, hazard ratio; Neff, effective sample size; P(better), probability of erdafitinib being better than EV; RR, risk ratio.
